# A dimeric mRNA vaccine based on key-mutant and KP.3 RBDs induces broad-spectrum immunity and protects mice against JN.1 and XDV challenges

**DOI:** 10.1186/s43556-026-00560-y

**Published:** 2026-09-03

**Authors:** Jiali Xu, Rui Peng, Longhai Yuan, Huan Zhang, Cong Tang, Yanan Zhou, Hao Yang, Yun Yang, Qing Huang, Junbin Wang, Hongyu Chen, Wenqi Quan, Baisheng Li, Qiangming Sun, Shuaiyao Lu

**Affiliations:** 1 Institute of Medical Biology, Chinese Academy of Medical Sciences & Peking Union Medical College, Kunming, China; 2 Guangdong Provincial Center for Disease Control and Prevention, Guangzhou, China; 3 Yunnan Key Laboratory of Cross-Border Infectious Disease Control and Prevention and Novel Drug Development, Yunnan University, Kunming, China; 4 State Key Laboratory of Respiratory Health and Multimorbidity, Beijing, China; 5 Key Laboratory of Pathogen Infection Prevention and Control (Peking Union Medical College), Ministry of Education, Beijing, China; 6 Yunnan Provincial Key Laboratory of Vector-Borne Diseases Control and Research, Kunming, China

**Keywords:** SARS-CoV-2, MRNA vaccine, KP.3, XDV, Broad-spectrum protection

## Abstract

The continued antigenic evolution of SARS-CoV-2 Omicron subvariants has progressively eroded vaccine-elicited protective immunity, driving demand for next-generation candidates that confer broad-spectrum protection against phylogenetically divergent strains. Here we report the design and preclinical evaluation of SV, an mRNA vaccine encoding a heterodimeric receptor-binding domain (RBD) antigen. In this construct, a previously optimized monomeric RBD (BSCOV06) is tandemly linked to the KP.3 RBD, presenting two antigenically distinct RBDs within a single immunogen. A two-dose SV regimen in BALB/c mice elicited high-titer neutralizing antibodies with potent cross-reactivity against BA.1, XBB.1.5, JN.1, KP.3, and the phylogenetically distant XDV variant. Integrated B cell receptor (BCR) and T cell receptor (TCR) repertoire profiling revealed that SV drives qualitatively distinct adaptive immune remodeling relative to BSCOV06. Key features included elevated class-switched somatic hypermutation, sustained naive B cell engagement, broad polyclonal T cell expansion, and extensive VJ gene-usage reprogramming across both lymphocyte compartments. In BALB/c and K18-hACE2 transgenic mice, SV conferred robust protection against JN.1 and XDV challenge, substantially reducing pulmonary viral loads and attenuating histopathological injury. Notably, SV achieved immunogenicity and cross-protective efficacy comparable to or exceeding those of the three-dose BSCOV06 schedule, supporting the potential of heterodimeric antigen design. These findings support SV as a promising broad-spectrum COVID-19 vaccine candidate. More broadly, they suggest that heterodimeric RBD architectures incorporating antigenically divergent variants may represent a generalizable platform for countering viral immune evasion, with implications for future SARS-CoV-2 variants and other rapidly evolving viral pathogens.

**Supplementary Information:**

The online version contains supplementary material available at https://doi.org/10.1186/s43556-026-00560-y.

## Introduction

The coronavirus disease 2019 (COVID-19) pandemic, caused by severe acute respiratory syndrome coronavirus 2 (SARS-CoV-2), has resulted in over 770 million confirmed cases and 7.1 million deaths globally as of November 2025 [1]. Vaccination remains the cornerstone of pandemic control, yet the continuous antigenic evolution of SARS-CoV-2 has progressively eroded vaccine-elicited immunity [2–6]. The Omicron lineage and its descendants—most notably the JN.1 subvariant and its progeny KP.2, KP.3, and XDV—have accumulated a dense array of immune-evasive mutations within the receptor-binding domain (RBD) [7–9]. Among these variants, KP.3 stands out: a unique epistatic interaction between the F456L and Q493E mutations simultaneously enhances ACE2 binding affinity and confers profound resistance to class 1 neutralizing antibodies [10–14], giving KP.3 a fitness and immune escape advantage over both JN.1 and KP.2 [15]. The World Health Organization has accordingly recommended updating vaccine antigen compositions to JN.1-lineage sequences [16, 17], creating an urgent need for next-generation vaccines that incorporate KP.3-derived RBD antigens.

The RBD of the spike protein is the principal target of neutralizing antibodies and an established immunogen for vaccine design [18, 19]. RBD-based antigens focus the humoral response on critical neutralizing epitopes while minimizing exposure to non-neutralizing epitopes elsewhere on the spike protein [20, 21]. Heterodimeric RBD architectures, which present two antigenically distinct RBDs in tandem, elicit broader and more potent neutralizing responses than their homodimeric or monomeric counterparts, likely by expanding the repertoire of B cell clones recruited during affinity maturation [22, 23]. mRNA-lipid nanoparticle (mRNA-LNP) platforms are well suited to engaging both humoral and cellular arms of adaptive immunity and offer rapid, iterative antigen redesign, intrinsic adjuvanticity, and a well-established clinical safety profile [24–26].

We previously engineered BSCOV06, a monomeric RBD-based mRNA vaccine incorporating H346T, L368I, V445P, and F490S mutations, which conferred robust immunogenicity against XBB and EG.5 sub-lineages [27, 28]. However, its monomeric design and pre-JN.1 RBD backbone limit its neutralization breadth against KP.3-derived variants. Moreover, how vaccine-induced B cell receptor (BCR) and T cell receptor (TCR) clonal architecture contributes to the breadth of RBD vaccines remains poorly characterized. We hypothesized that a heterodimeric RBD antigen—composed of the BSCOV06 RBD and the KP.3 variant RBD covalently linked in tandem—would elicit superior humoral and cellular immunity while driving qualitatively distinct B and T cell repertoire remodeling relative to the monomeric prototype.

To test this hypothesis, we designed SV, an mRNA-LNP vaccine encoding a BSCOV06–KP.3 heterodimeric RBD antigen, and evaluated it in two mouse models. In BALB/c mice, two-dose SV vaccination elicited high-titer cross-neutralizing antibodies against BA.1, XBB.1.5, JN.1, KP.3, and the antigenically distant XDV variant, and induced robust germinal center B cell expansion, effector and central memory T cell differentiation, and a Th1-skewed cytokine profile. Integrated BCR and TCR repertoire profiling further revealed that SV drives elevated class-switched somatic hypermutation, sustained naive B cell engagement, broad polyclonal T cell expansion, and extensive VJ gene-usage reprogramming relative to BSCOV06. In K18-hACE2 transgenic mice, SV conferred robust protection against challenge with JN.1 and XDV, substantially reducing pulmonary viral loads and attenuating histopathological injury. These findings support SV as a promising broad-spectrum COVID-19 vaccine candidate and suggest that heterodimeric RBD designs incorporating antigenically divergent RBDs may represent an effective strategy for broadening vaccine-elicited immunity against evolving SARS-CoV-2 variants.

## Results

### Characterization, expression, and biodistribution of the dimeric antigen mRNA vaccine SV

**Fig. 1:**
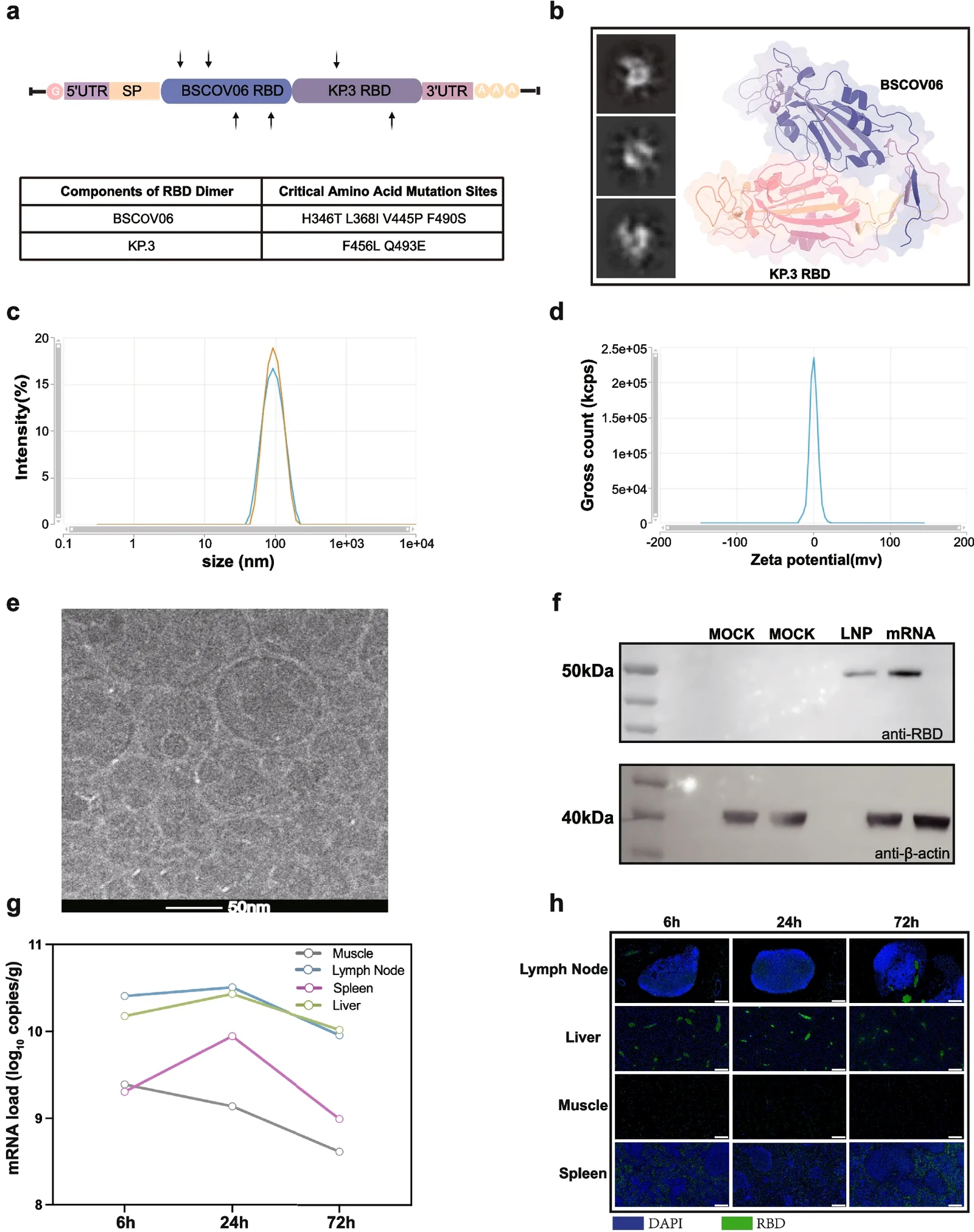
Characterization, expression, and biodistribution of the dimeric antigen mRNA vaccine SV. **a** Construction strategy and key mutation sites of the SV antigen. **b** Two-dimensional survey diagram and predicted structure of the heterodimer. **c**, **d** Physicochemical characterization of the synthesized mRNA-LNP formulation, illustrating the hydrodynamic diameter (**c**) and zeta potential (**d**) determined by dynamic light scattering (DLS). **e** Representative cryo-electron microscopy (Cryo-EM) image showing the morphology and internal structure of the mRNA-LNPs. **f** Western blot analysis confirming the expression and molecular weight of the target antigen. **g** Antigen mRNA abundance across tissues at serial time points following vaccination. **h** Immunofluorescence detection of antigen expression across tissues at serial time points following vaccination (Scale bar, 100 μm)

To address the waning efficacy of existing vaccines against emerging SARS-CoV-2 variants, we sought to develop a broad-spectrum mRNA vaccine. We previously showed that incorporating key immune-escape sites from multiple variants into the ancestral (Wuhan-Hu-1) RBD enhanced the cross-protective efficacy of a subunit vaccine [29]. Building on this, we introduced additional mutations to generate the candidate antigen BSCOV06 [30]. We then engineered a heterodimeric antigen, designated SV (Fig. 1a), as a single-chain fusion protein in which the KP.3 variant RBD is fused to the BSCOV06 scaffold, to improve neutralization of currently dominant lineages.

Cryo-EM 2D classification was consistent with a dimer-like morphology, and the protein structure was predicted in silico with AlphaFold3 (Fig. 1b). Immune epitope prediction suggested that SV harbors abundant B-cell and MHC epitopes, indicating its potential to elicit favorable immunogenicity (Fig. S1). These structural and epitope-level analyses are computational in nature and await experimental verification. The IVT mRNA was encapsulated into LNPs by microfluidic mixing. Dynamic light scattering (DLS) gave a mean particle size of 102.3 ± 3.5 nm with a polydispersity index (PDI) of 0.08, indicating a monodisperse population. The zeta potential was −28.4 ± 1.2 mV (Fig. 1c, d), and encapsulation efficiency exceeded 95%. Cryo-EM confirmed the spherical, monodisperse morphology (Fig. 1e). These data show that the SV mRNA-LNP formulation is stable and homogeneous. Western blotting verified expression of the SV antigen in HEK 293 T cells (Fig. 1f).

To characterize biodistribution and in vivo stability, we collected lymph node, muscle, liver, and spleen tissues at 6, 24, and 72 h after intramuscular injection and assessed both RNA distribution and antigen expression. RNA and protein were detectable in multiple tissues, including lymph nodes (Fig. 1g, h), consistent with the intrinsic tropism of conventional four-component LNPs after intramuscular delivery. We also verified mRNA integrity and in vivo stability at serial time points by reverse transcription, PCR, and agarose gel electrophoresis (Fig. S2). Together, these results confirm the successful construction of a stable mRNA-LNP vaccine system.

### SV vaccine elicits self-limiting innate activation and broad-spectrum neutralizing antibody responses

**Fig. 2:**
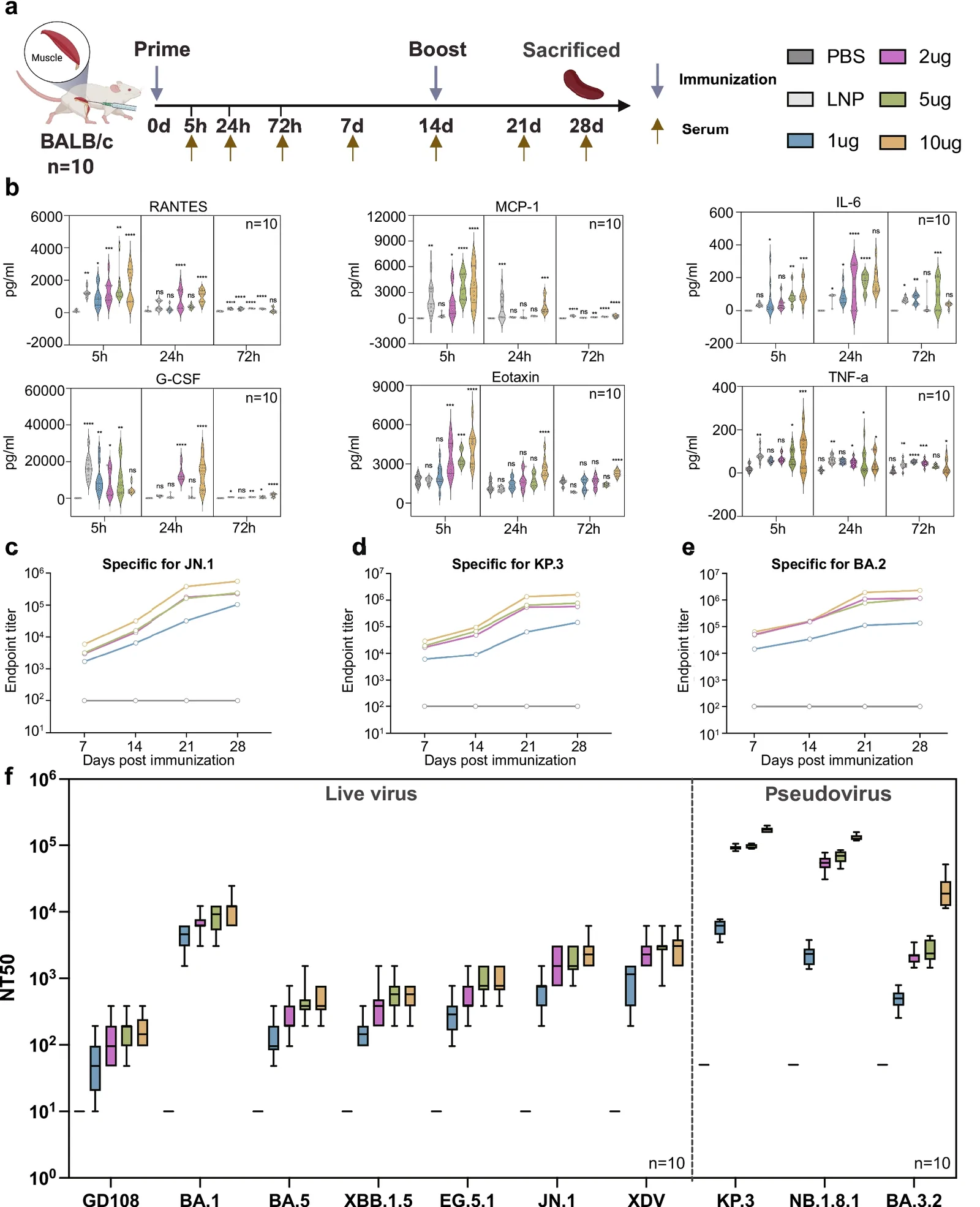
SV vaccine elicits self-limiting innate activation and broad-spectrum neutralizing antibody responses. **a** Schematic illustration of the immunization schedule and sampling timeline in BALB/c mice (created with BioRender.com). **b** Expression of RANTES, G-CSF, MCP-1, Eotaxin, IL-6, and TNF-α at 5, 24, and 72 h post-immunization (*n* = 10). **c** RBD-specific binding antibody titers against JN.1 in BALB/c mouse sera (*n* = 10). **d** RBD-specific binding antibody titers against KP.3 in BALB/c mouse sera (*n* = 10). **e** RBD-specific binding antibody titers against BA.2 in BALB/c mouse sera (*n* = 10). **f** Neutralizing antibody titers against diverse SARS-CoV-2 variants in the sera of immunized BALB/c mice (*n* = 10). Statistical analysis: Data were analyzed using one-way ANOVA followed by Dunnett’s test. Significance is indicated as: *P < 0.05; **P < 0.01; ***P < 0.001; ****P < 0.0001; ns, not significant

To comprehensively evaluate the immunostimulatory capacity and immunogenicity of SV, BALB/c mice were immunized intramuscularly with different doses of mRNA-LNP, empty LNPs (without mRNA), or PBS as a control. Serum samples were collected at 5 h, 24 h, 72 h, and on days 7, 14, 21, and 28 post-immunization (Fig. 2a). Analysis of the dynamic changes in multiple serum cytokines revealed that, compared with the PBS group, the levels of IL-6, TNF-α, MCP-1, RANTES, G-CSF, and Eotaxin were elevated to varying degrees at 5 h, followed by a progressive decline at 24 h and 72 h (Fig. 2b). These results indicate that both empty LNPs and mRNA-LNPs rapidly activate innate immunity and promote immune cell recruitment without inducing sustained inflammation, suggesting a self-limiting innate immune response.

We subsequently evaluated the kinetics of antigen-specific binding IgG antibodies across different groups. Overall, immunization with mRNA-LNP elicited specific IgG responses against JN.1, KP.3, and BA.2 (Fig. 2c–e), with titers reaching the highest level at day 21 post-prime among the time points examined. Specifically, the 10 μg group produced the highest IgG titers (~10⁶), while the 1 μg group yielded the lowest (~10^5^), showing a clear dose-dependent response. In contrast, the 2 μg and 5 μg groups generated comparable antibody levels (between 10^5^ and 10⁶), exhibiting no overt dose dependency. Neutralizing antibody titers against various strains, measured at day 14 post-boost using either authentic virus or pseudovirus neutralization assays, displayed a dose–response pattern consistent with that of the binding IgG data (Fig. 2f). Among the authentic virus neutralization results, the highest neutralizing titers were observed against BA.1 (~10^4^), whereas the lowest were directed against the original strain (~10^2^), representing a 100-fold difference (Fig. S3). Notably, in addition to eliciting neutralizing antibodies against the strains used for antigen design (KP.3 and its ancestral lineages), the SV vaccine also induced appreciable levels of neutralizing antibodies against NB.1.8.1, BA.3.2, and XDV. These results indicate that SV elicits a robust humoral immune response against a broad range of strains. Furthermore, this broad-spectrum neutralizing activity persisted up to 240 days after prime immunization, consistent with the establishment of humoral immune memory and potential long-term protective efficacy (Fig. S4). However, because formal kinetic and booster-response experiments were not performed, these data do not establish the optimal booster schedule.

### SV vaccination elicits robust GC reactions, establishes cellular immune memory, and drives Th1-skewed immunity

**Fig. 3:**
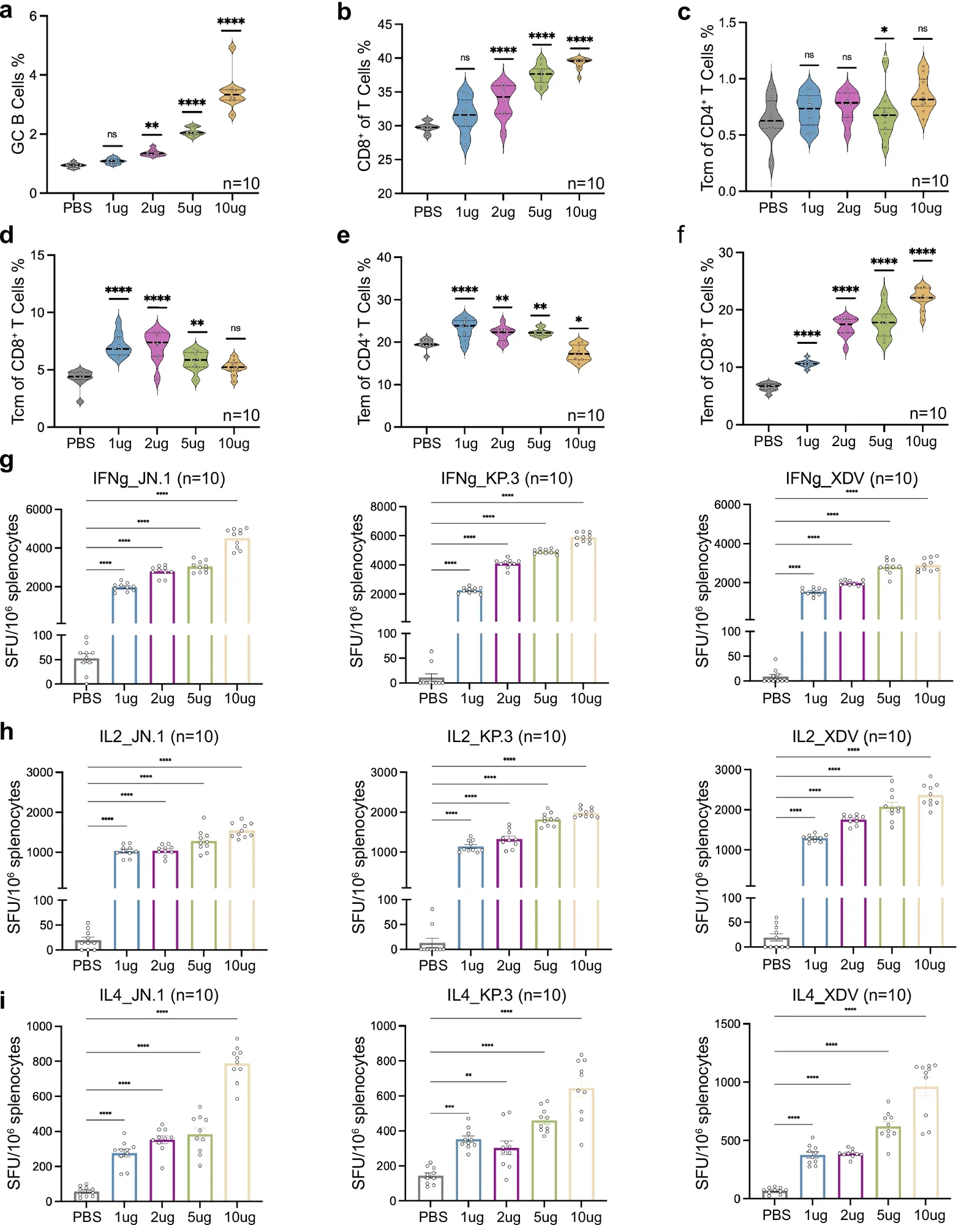
SV vaccination elicits robust GC reactions, establishes cellular immune memory, and drives Th1-skewed immunity. **a** Frequency of splenic germinal center B cells after vaccination. **b** Frequency of splenic CD8⁺ T cells after vaccination. **c**–**f** Frequencies of effector memory T cells (Tem) and central memory T cells (Tcm) in the CD4⁺ and CD8⁺ compartments after vaccination. **g–i** Quantification of antigen-specific cytokine-secreting splenocytes harvested from BALB/c mice (*n* = 10). Splenocytes were stimulated with recombinant JN.1 RBD protein, KP.3 RBD protein and XDV protein to evaluate the secretion levels of IFN-γ (**g**), IL-2 (**h**), and IL-4 (**i**). Results are expressed as the number of spot-forming unit (SFU) per million splenocytes. Statistical analysis: Data were analyzed using one-way ANOVA followed by Dunnett’s test. Significance is indicated as: *P < 0.05; **P < 0.01; ***P < 0.001; ****P < 0.0001; ns, not significant

To evaluate the immune mechanisms of the SV vaccine, we collected spleens at 14 days post-boost and performed flow cytometric analysis (Fig. S5). SV vaccination significantly expanded germinal center (GC) B cells (Fig. 3a), indicating productive germinal center reactions that support antibody affinity maturation and broadly neutralizing antibody generation. SV also promoted CD8⁺ T cell proliferation (Fig. 3b), consistent with the initiation of antigen-specific cytotoxic T cell responses. We next examined central memory T cells (Tcm) and effector memory T cells (Tem) within splenic CD4⁺ and CD8⁺ compartments. SV predominantly expanded CD8⁺ Tcm, whereas CD4⁺ Tcm remained largely unchanged (Fig. 3c, d), suggesting that SV-induced CD8⁺ memory possesses long-term central reserve capacity while CD4⁺ memory is preferentially polarized toward the peripheral effector compartment. SV expanded both CD4⁺ Tem and CD8⁺ Tem populations, indicating the establishment of peripheral immune surveillance capable of mounting rapid recall responses upon antigen re-exposure (Fig. 3e, f).

Cellular immunity is essential for antiviral defense. To quantify the vaccine-induced cellular immune response, we isolated splenocytes from BALB/c mice at 14 days post-boost and performed ELISpot assays using JN.1 RBD, KP.3 RBD, and XDV RBD proteins as stimulatory antigens (Fig. 3g–i, Fig. S6). All three cytokines were significantly elevated, with IFN-γ and IL-2 spot-forming units exceeding those of IL-4. IFN-γ, a core antiviral effector, reflects robust antiviral activity, while IL-2 drives effector T cell clonal expansion and sustains memory T cell survival, indicating that SV activates both Th1 and Th2 responses while establishing a Th1-skewed, antigen-specific cellular immune response conducive to antiviral defense.

### SV confers cross-protection against JN.1 and XDV in BALB/c and K18-hACE2 mice

**Fig. 4:**
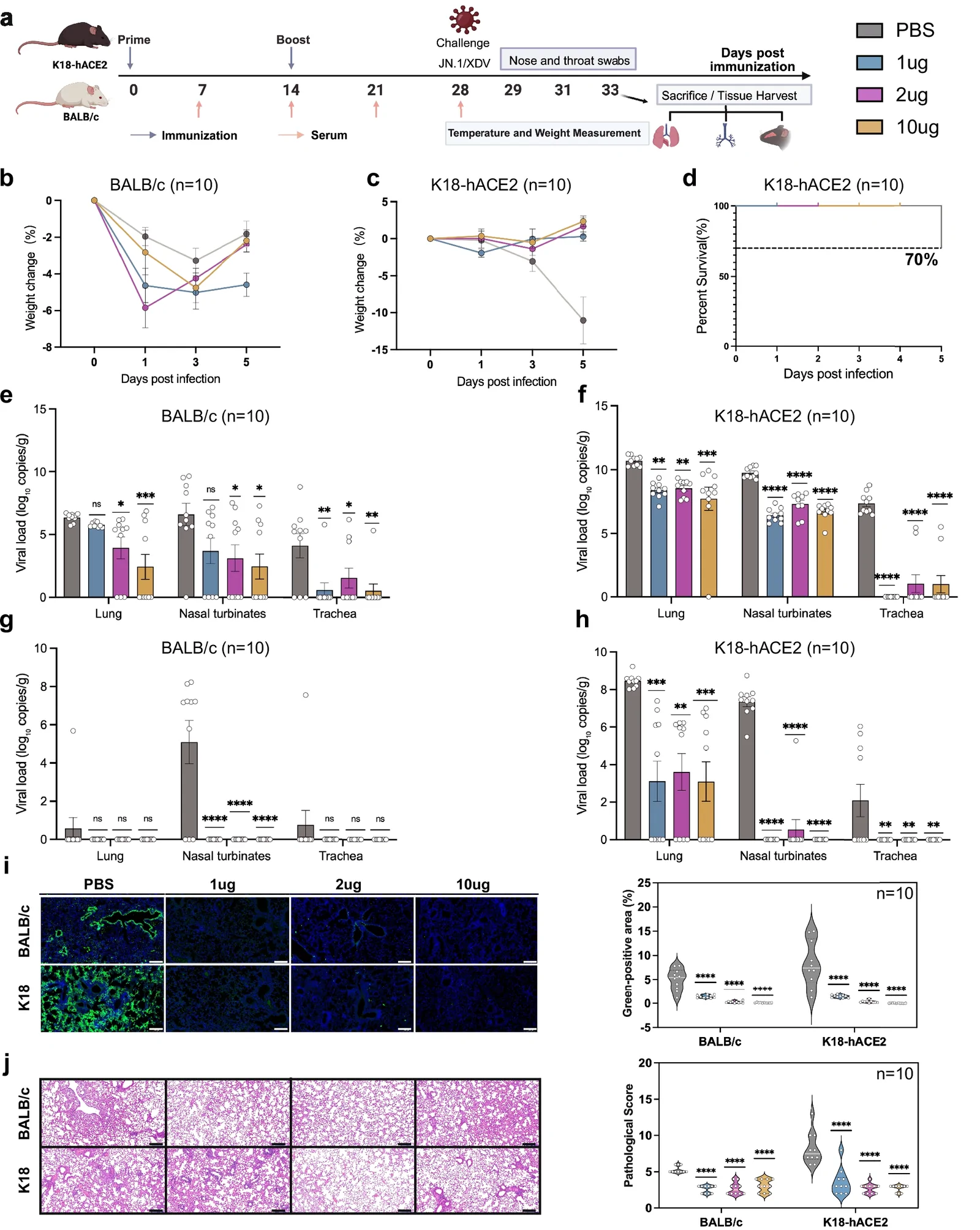
SV confers cross-protection against JN.1 and XDV in BALB/c and K18-hACE2 mice. **a** Schematic of the immunization and challenge experimental design for SV vaccine evaluation in BALB/c and K18-hACE2 mice (created at BioRender.com). **b** Body weight changes in different groups of BALB/c mice over 5 days after XDV infection (*n* = 10). **c** Body weight changes in different groups of K18-hACE2 mice over 5 days after XDV infection (*n* = 10). **d** Survival curves of different groups of K18-hACE2 mice over 5 days after XDV infection (*n* = 10). **e** Pulmonary gRNA loads in different groups of BALB/c mice at 5 days after XDV infection (*n* = 10). **f** Pulmonary gRNA loads in different groups of K18-hACE2 mice at 5 days after XDV infection (*n* = 10). **g** Pulmonary sgRNA loads in different groups of BALB/c mice at 5 days after XDV infection (*n* = 10). **h** Pulmonary sgRNA loads in different groups of K18-hACE2 mice at 5 days after XDV infection (*n* = 10). **i** Expression of viral N antigen in the lungs of different groups of BALB/c and K18-hACE2 mice at 5 days after XDV infection (*n* = 10, scale bar, 200 μm). **j** Lung histopathology and pathological scores in different groups of BALB/c and K18-hACE2 mice at 5 days after XDV infection (*n* = 10, scale bar, 200 μm). Statistical analysis: Data were analyzed using one-way ANOVA followed by Dunnett’s test. Significance is indicated as: *P < 0.05; **P < 0.01; ***P < 0.001; ****P < 0.0001; ns, not significant

To evaluate the protective efficacy of the SV vaccine, we performed challenge experiments using the XDV strain (an antigenically distant variant within the Omicron lineage) and the JN.1 strain in BALB/c and K18-hACE2 transgenic mouse models. BALB/c mice were challenged using a two-dose immunization regimen, whereas K18-hACE2 mice received the same prime-boost immunization regimen before challenge (Fig. 4a). In the K18-hACE2 model, SV immunization induced binding antibody responses, neutralizing antibody responses, and antigen-specific cellular immunity, and its immunogenicity profile was consistent with observations in BALB/c mice (Fig. S7). In BALB/c mice, no group showed significant changes in body weight or body temperature after XDV challenge (Fig. 4b). In K18-hACE2 mice, animals in the PBS control group exhibited rapid body weight loss during the 5-day monitoring period after XDV infection, with a survival rate of only 70%, whereas mice in the SV-vaccinated group maintained stable body weight and achieved 100% survival (Fig. 4c, d). Furthermore, in both mouse models, viral loads in nasopharyngeal swab samples from vaccinated mice infected with the XDV strain were markedly lower than those in the PBS group, indicating that the vaccine-induced immune response can effectively suppress viral replication locally in the upper respiratory tract mucosa (Fig. S8).

Subsequently, we collected lung, trachea, and nasal turbinate tissues from each group of mice on day 5 after XDV challenge for viral load measurement, and we measured gRNA and sgRNA respectively (*n* = 10; Fig. 4e–h). The measurement results showed that, compared with the control group, viral replication levels in the lungs and other tissues of vaccinated mice were significantly reduced and showed a dose-dependent pattern. Notably, the 10 μg dose group achieved clearance of sgRNA in both mouse models, indicating that SV can effectively suppress viral replication in the lungs, nasal turbinates, and trachea. In addition, we performed TCID_50_, immunofluorescence, and histopathological analyses on the lungs of mice in each group. For TCID_50_ (*n* = 5), specific values were detected only in the PBS group of K18-hACE2 mice, while the vaccine groups showed significantly reduced levels, all below the detection limit (Fig. S8). Immunofluorescence staining for SARS-CoV-2 nucleocapsid (N) protein showed markedly attenuated signals in the lungs of SV-vaccinated animals in both models (Fig. 4i). Histopathological analysis further validated these results: compared with the control group, lung lesion scores in SV-vaccinated mice were significantly reduced, and lung injury (including inflammatory infiltration, alveolar destruction, hemorrhage, and vascular thrombosis) was substantially alleviated (Fig. 4j).

We conducted a challenge protection evaluation of the JN.1 strain following the same protocol described above (*n* = 5). The results showed that all vaccine groups exhibited similar protective efficacy in BALB/c and K18-hACE2 mice, with good protective effects against basic clinical signs of viral infection, viral load, and pathological damage (Fig. S9). Overall, the challenge protection results indicated that two-dose SV vaccination can confer cross-protection against both JN.1 and XDV strains in immunocompetent and humanized mouse models, as evidenced by reduced pulmonary viral load, suppressed active viral replication, and alleviated histopathological injury, among others (Fig. S10).

### SV vaccination elicits qualitatively distinct B and T cell repertoires relative to BSCOV06

We previously found that two immunizations with SV elicited humoral and cellular responses matching or exceeding those induced by the prototype BSCOV06 vaccine. To investigate the receptor repertoire features associated with this enhanced immunogenicity, we performed paired BCR and TCR sequencing on peripheral blood from mice receiving two doses of SV or BSCOV06, with PBS-injected mice as negative controls.

**Fig. 5:**
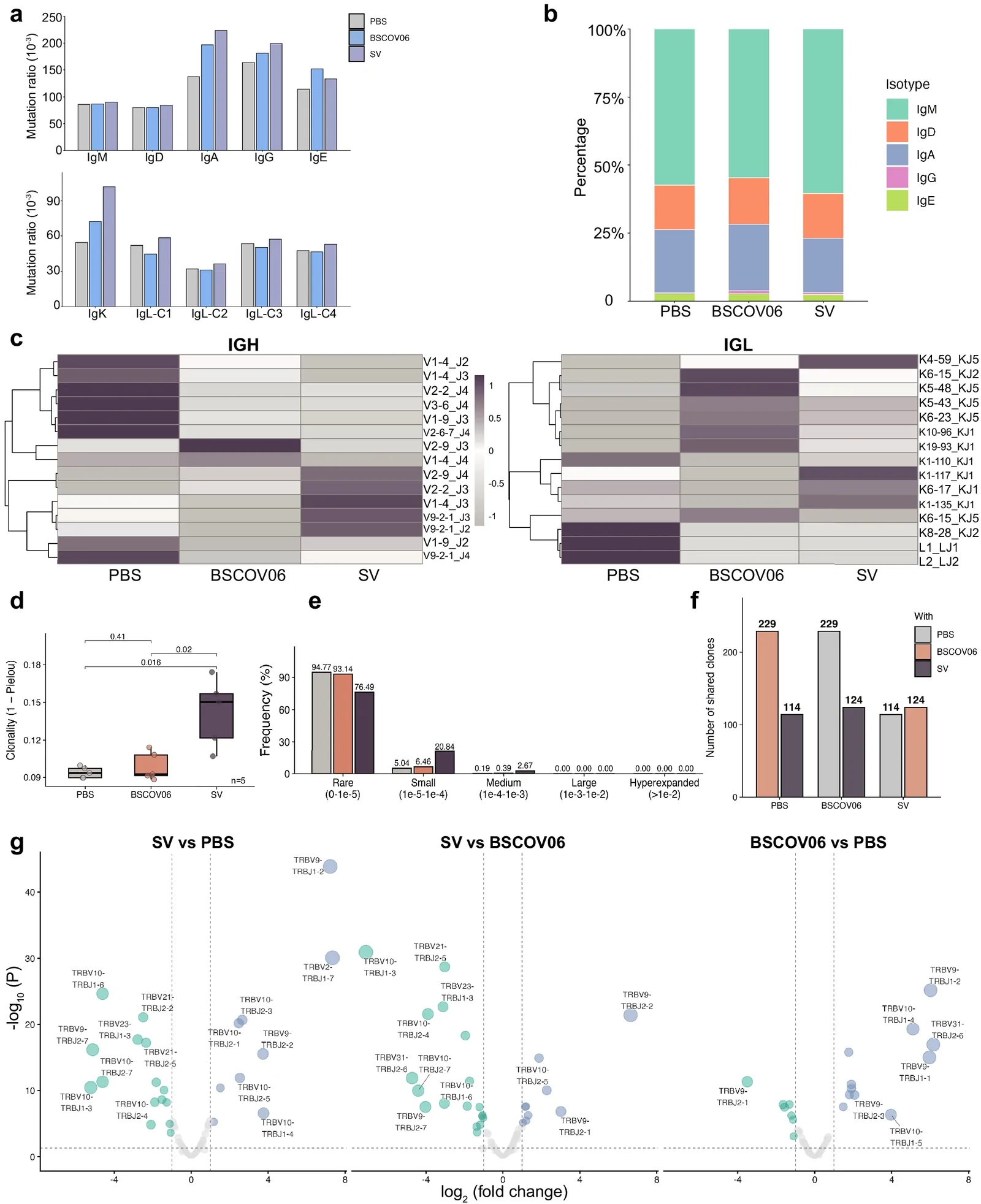
SV vaccination elicits qualitatively distinct B and T cell repertoires relative to BSCOV06. **a** Somatic hypermutation (SHM) rates of BCR IGH and IGL variable genes (*n* = 5). **b** IGH isotype distribution by group (*n* = 5). **c** Heatmap of the top 15 most abundant VJ gene pairs in IGH (left) and IGL (right), colored by z-score of group-level frequency. **d** TCRβ clonality (1 − Pielou evenness) across groups (*n* = 5). **e** Clone size hierarchy of the TCRβ repertoire. **f** Number of shared TCRβ clonotypes between groups. **g** Volcano plots of differential TCRβ VJ pair usage for all three pairwise comparisons. Statistical analysis: Data were analyzed using one-way ANOVA followed by Dunnett’s test. Significance is indicated as: *P < 0.05; **P < 0.01; ***P < 0.001; ****P < 0.0001; ns, not significant

In the BCR compartment, SV-vaccinated mice showed markedly higher somatic hypermutation (SHM) rates in class-switched IGH isotypes than either control group. IgA SHM reached 223.5 per 10^3^ bp in SV, compared with 196.9 in BSCOV06 and 137.3 in PBS; IgG SHM was similarly elevated (199.5 vs 181.2 and 163.9), and the Igκ (IGK) chain exhibited the same pattern (101.9 vs 72.2 and 54.6) (Fig. 5a). Despite this enhanced SHM, the SV group retained the highest proportion of unswitched IgM (60.4% vs 54.6% in BSCOV06 and 57.3% in PBS), suggesting that SV simultaneously promotes affinity maturation in class-switched compartments while sustaining ongoing naïve B cell recruitment — a feature absent in BSCOV06 (Fig. 5b). Hierarchical clustering of the top 15 most abundant VJ gene pair combinations separated SV samples from both BSCOV06 and PBS controls across IGH and IGL chains. In the IGL compartment, SV exceeded BSCOV06 in both Shannon diversity (7.35 vs 7.17) and unique clone count (179,215 vs 110,678), reflecting a more diversified light chain repertoire (Fig. 5c). Differential VJ usage analysis identified the pairings driving this divergence: in IGH, IGHV3S1–IGHJ2, IGHV3S1–IGHJ3, and IGHV7-4–IGHJ3 were strongly SV-biased; in IGL, IGKV6-d–IGKJ2, IGKV2-a–IGKJ5, and IGKV12-e–IGKJ1 were selectively enriched in SV, with several pairings undetectable in PBS controls.

In the TCR compartment, the contrast between SV and BSCOV06 was more pronounced. SV induced significantly higher TCRβ clonality than BSCOV06 (mean 0.142 vs 0.099), whereas BSCOV06 was indistinguishable from PBS (0.094, *P* = 0.41) (Fig. 5d). This elevated clonality reflected broad polyclonal expansion, not oligoclonal dominance: the Rare clone fraction (frequency below 10^−5^) fell from 93.1% in BSCOV06 to 76.5% in SV, with corresponding gains in the Small and Medium categories (Fig. 5e). The SV repertoire was the most immunologically distinct: SV shared only 124 TCRβ clonotypes with BSCOV06, whereas BSCOV06 shared 229 clonotypes with PBS, indicating that SV vaccination elicits a T cell repertoire that diverges substantially from the baseline response generated by the prototype vaccine (Fig. 5f). Differential VJ pair analysis confirmed this divergence: the top SV-enriched pairings versus BSCOV06 were TRBV9–TRBJ2-2 and TRBV9–TRBJ2-1, and SV-enriched pairings versus PBS were led by TRBV2–TRBJ1-7 and TRBV9–TRBJ1-2 (Fig. 5g).

Together, these data indicate that SV vaccination engages a qualitatively distinct adaptive immune program that exceeds BSCOV06 in at least four dimensions: enhanced SHM without sacrificing naïve B cell reserves, greater light chain diversification, broader polyclonal T cell expansion, and extensive VJ repertoire remodeling across both B and T cell compartments. These coordinated features correlate with the enhanced immunogenicity of SV observed in vivo; however, the molecular mechanism by which the fused-antigen design drives these repertoire changes remains to be established.

### SV vaccination exhibits a favorable preliminary safety profile in BALB/c mice

**Fig. 6:**
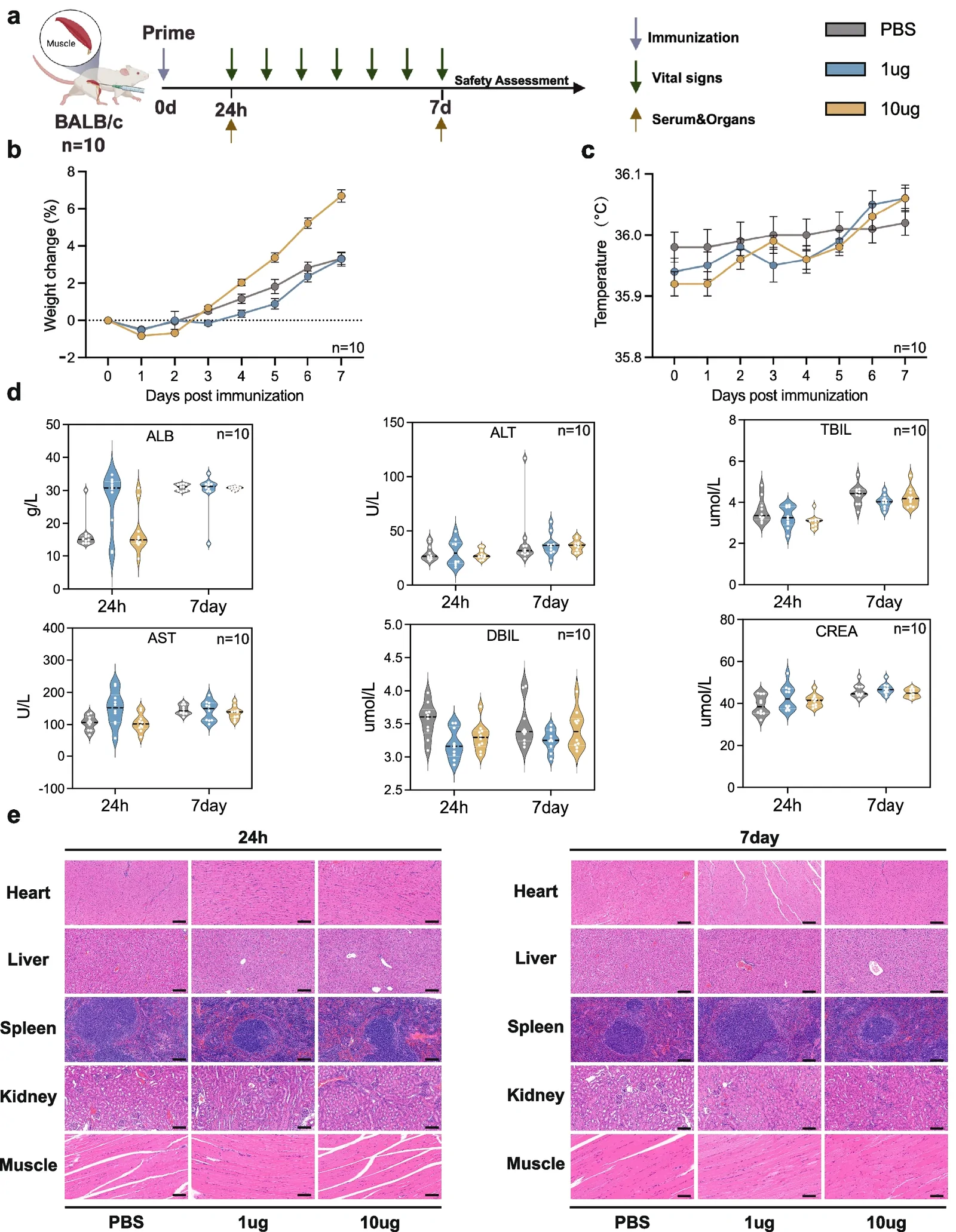
SV vaccination exhibits a favorable preliminary safety profile in BALB/c mice. **a** Schematic of the SV vaccine preliminary safety evaluation workflow (Created with BioRender). **b** Body weight changes in BALB/c mice within 7 days post-immunization (*n* = 10). **c** Body temperature changes in BALB/c mice within 7 days post-immunization (*n* = 10). **d** Serum biochemical parameters in BALB/c mice at 24 h and 7 days post-immunization (*n* = 10). **e** Histopathological analysis of major organs and tissues in BALB/c mice at 24 h and 7 days post-immunization (*n* = 10). Statistical analysis: Data were analyzed using one-way ANOVA followed by Dunnett’s test. Significance is indicated as: *P < 0.05; **P < 0.01; ***P < 0.001; ****P < 0.0001; ns, not significant

To evaluate the safety profile of the SV vaccine, we conducted a preliminary safety assessment in BALB/c mice. As shown in Fig. 6a, mice were immunized with a low dose (1 μg) or a high dose (10 μg) of the SV vaccine, with PBS serving as the control, and basic physiological parameters—including body weight and body temperature—were monitored daily from day 1 to day 7 post-immunization. Blood and tissue samples were collected at 24 h and on day 7 to preliminarily assess the impact of the SV vaccine on biochemical parameters and tissue integrity. Throughout the 7-day observation period, neither body weight (Fig. 6b) nor body temperature (Fig. 6c) was markedly affected. Serum biochemical indicators reflecting hepatocyte integrity (ALT, AST), hepatobiliary excretory function (TBIL, DBIL), hepatic synthetic function (ALB), and renal function (CREA) showed no notable abnormalities at either 24 h or day 7 compared with the PBS control group (Fig. 6d), providing preliminary evidence that the SV vaccine does not induce appreciable hepatic or renal toxicity, nor does it adversely affect hepatic synthetic or metabolic function. Consistently, histopathological analysis of multiple tissues and organs, including muscle and liver, at 24 h and day 7 (Fig. 6e) revealed no vaccine-induced pathological alterations. Collectively, these preliminary safety data indicate that the SV vaccine possesses a favorable safety profile in BALB/c mice.

## Discussion

The continuous antigenic drift of the SARS-CoV-2 Omicron lineage has eroded the protective efficacy of strain-matched, monomeric vaccines, underscoring the need for next-generation strategies with improved breadth and potency [7–9, 13–15]. Heterodimeric RBD vaccines, which present two antigenically distinct RBDs in tandem, have been shown to elicit broader neutralizing responses than their homodimeric or monomeric counterparts [22, 31, 32]. However, whether this approach—when adapted to the JN.1/KP.3 lineage—confers advantages beyond improved serological endpoints has not been systematically examined.

In this study, we compared SV, a heterodimeric RBD mRNA vaccine linking the BSCOV06 RBD with the KP.3 variant RBD, against the monomeric BSCOV06 prototype. The central finding is that two-dose SV immunization achieved binding and neutralizing antibody titers, antigen-specific T cell responses, and in vivo cross-protection that were comparable to or exceeded those of three-dose BSCOV06 in both BALB/c and K18-hACE2 mouse models. This dose-sparing effect may be of practical relevance, as reduced immunization schedules could improve compliance and facilitate outbreak response; however, direct comparisons with currently authorized mRNA vaccines were not performed.

To investigate potential correlates of this enhanced potency, we compared the antibody profiles, cellular immune responses, and BCR/TCR repertoire features of SV and BSCOV06. At the humoral level, SV elicited high-titer cross-neutralizing antibodies against a broad panel of variants, including BA.1, XBB.1.5, JN.1, KP.3, and the antigenically distant XDV variant. At the cellular level, ELISpot assays revealed that SV induced robust antigen-specific IFN-γ, IL-2, and IL-4 secretion, with IFN-γ and IL-2 predominating over IL-4—a Th1-skewed cytokine profile conducive to antiviral defense. At the repertoire level, SV vaccination was associated with elevated class-switched somatic hypermutation, higher IGL diversity, broader polyclonal TCRβ expansion, and extensive VJ gene-usage divergence relative to BSCOV06. These repertoire features are correlative, consistent with the enhanced serological and cellular responses, and suggest that the heterodimeric architecture engages the adaptive immune system in a qualitatively more diversified manner than the monomeric design.

SV differs from earlier heterodimeric RBD vaccines in a key respect: rather than pairing two temporally adjacent RBDs, it couples the BSCOV06 RBD backbone with the KP.3 RBD, which harbors the F456L/Q493E epistatic pair responsible for the enhanced fitness of current JN.1-lineage variants [15, 17, 18]. It is plausible that this deliberate antigenic updating, combined with the heterodimeric format, contributes to the breadth of cross-neutralization observed. However, the relative contributions of the dimeric architecture versus the specific KP.3 mutations cannot be disentangled from the current data and would require head-to-head comparison with an updated monomeric KP.3-based vaccine.

Although we have performed a preliminary evaluation of the immunogenicity, protective efficacy, immune mechanisms, and safety of the SV vaccine, several limitations remain. In vivo protection efficacy and preliminary safety profile were assessed in BALB/c or K18-hACE2 mouse models, and evaluation in non-human primates is needed to support clinical translation. Protection was demonstrated against JN.1 and XDV, but efficacy against variants emerging after the study period remains untested. The current mRNA-LNP formulation primarily elicits systemic immunity; mucosal immune engagement may require optimized delivery strategies [33–37]. Furthermore, the structural and epitope-level evidence for SV is computational in nature and awaits confirmation by experimental high-resolution structural determination. In addition, because formal kinetic and booster-response experiments were not performed, the optimal booster schedule has not yet been established. Moreover, the immunization dose was selected on the basis of our preclinical dose-titration experiments, and formal pharmacokinetic/pharmacodynamic (PK/PD) studies were not conducted; the dose used here should therefore be regarded as a preclinical dose rather than a clinical dose. Finally, this study is limited to comparison with the BSCOV06 prototype and does not include comparisons with currently used or updated mRNA vaccines (e.g., XBB.1.5-based vaccines), which to some extent constrains the assessment of the immunological advantages of the SV vaccine. These limitations will be addressed in future studies of the SV vaccine.

In summary, this study demonstrates that a heterodimeric RBD mRNA vaccine incorporating the KP.3 variant RBD can achieve two-dose immunogenicity and protection comparable to or exceeding those of the three-dose monomeric prototype BSCOV06, and exhibited a favorable preliminary safety profile. The antibody, cellular, and repertoire data collectively suggest that the heterodimeric design elicits qualitatively distinct immune responses beyond what serological endpoints alone capture. These findings support SV as a promising broad-spectrum COVID-19 vaccine candidate and illustrate a design approach that may be applicable to antigenically variable pathogens.

## Materials and methods

### Cells and viruses

Authentic SARS-CoV-2 variants (GD108, BA.1, BA.5, XBB.1.5, EG.5.1, JN.1, and XDV) utilized in this study were provided by the National Kunming High-level Biosafety Primate Research Center. All procedures involving live viruses were strictly conducted within biosafety level 3 (BSL-3) containment facilities. Vero and 293 T-hACE2 cells were maintained in Dulbecco’s Modified Eagle Medium (DMEM; Gibco, USA) supplemented with 10% heat-inactivated fetal bovine serum (FBS; Gibco, USA) and 1% penicillin–streptomycin (Gibco, USA) at 37 °C in a humidified incubator with 5% CO_2_.

### Experimental animals and ethical approval

Specific-pathogen-free (SPF), female BALB/c mice (6–8 weeks old) were purchased from the Institute of Medical Biology, Chinese Academy of Medical Sciences (IMBCAMS; permit number: SCXK (DIAN) 2022–0002). Transgenic female K18-hACE2 mice (6–8 weeks old) were obtained from GemPharmatech Co., Ltd. (permit number: SCXK (SU) 2023–0009). Mice were acclimated and housed in an SPF barrier facility under a standard 12 h light/dark cycle with free access to standard chow and water (ad libitum). For in vivo viral challenge studies, animals were transferred to and maintained within an animal biosafety level 3 (ABSL-3) facility. All animal care and handling procedures strictly complied with the Guide for the Care and Use of Laboratory Animals published by the National Institutes of Health (NIH).

### Preparation and characterization of the mRNA vaccine

In vitro transcription (IVT) was performed using the HiScribe T7 mRNA Kit (New England Biolabs, E2080S), with complete substitution of uridine-5′-triphosphate (UTP) by N1-methylpseudouridine-5′-triphosphate. The synthesized mRNA was purified and quantified before lipid nanoparticle (LNP) encapsulation. The lipid mixture — SM102, DMG-PEG 2000, DSPC, and cholesterol — was dissolved in anhydrous ethanol at a molar ratio of 50:1.5:10:38.5 for the organic phase. The purified mRNA was diluted in 50 mM sodium acetate buffer (pH 4.0) to 100–120 ng/μL as the aqueous phase. The aqueous and organic phases were mixed at a 3:1 volume ratio using a microfluidic mixer. Ethanol was removed and the buffer was exchanged into 20 mM Tris–HCl (pH 7.4) using 100 kDa molecular weight cut-off (MWCO) centrifugal filter units (Millipore, UFC9100). Encapsulation efficiency was measured using the Quant-iT RiboGreen RNA Assay Kit. Mean particle size, polydispersity index (PDI), and zeta potential were measured with a Zetasizer Pro (Malvern Panalytical). LNP morphology was examined by cryo-electron microscopy (cryo-EM) using a Talos F200C transmission electron microscope equipped with a Ceta 4 k × 4 k camera at Xi'an Jiaotong University.

### Immunization and viral challenge

To evaluate immunogenicity, healthy female BALB/c and K18-hACE2 mice were immunized intramuscularly (i.m.) with escalating doses (1, 2, 5, or 10 μg) of the candidate SV vaccine on days 0 and 14. Control groups received an equivalent volume of Tris–HCl buffer. Sera were collected at 7, 14, 21, and 28 days post-prime for quantification of RBD-specific binding antibody titers. Day 28 sera were additionally used for authentic virus neutralization assays. On day 28, splenocytes were isolated from parallel cohorts (*n* = 10) for assessment of cellular immune responses.

For in vivo efficacy assessment, immunized and placebo-control BALB/c and K18-hACE2 mice (*n* = 10/group) were challenged intranasally (i.n.) on day 28 post-prime with 1 × 10⁶ TCID_50_ of JN.1 or XDV. Nasopharyngeal swabs were collected at 1, 3, and 5 days post-infection (dpi) for viral load monitoring, and body weight and body temperature were recorded daily. At 5 dpi, mice were euthanized and respiratory tissues, including lungs, were harvested for viral load quantification and histopathological analysis.

### Viral load quantification and in vivo mRNA stability assay

Viral load was quantified in lung samples collected at 5 dpi. Tissues were homogenized in TRIzol reagent (Invitrogen, USA), and total RNA was extracted using an automated nucleic acid extraction system with a commercial kit (Biokeystone, M2006P-96KF). RT-qPCR was performed on a CFX384 Touch Real-Time PCR Detection System (Bio-Rad, USA) with the TaqMan Fast Viral 1-Step Master Mix (Thermo Fisher, 4,444,432). The primers and probe targeting SARS-CoV-2 were:

- forward primer, 5′-GACCCCAAAATCAGCGAAAT-3′;
- reverse primer, 5′-TCTGGTTACTGCCAGTTGAATCTG-3′;
- probe, 5′-FAM-ACYCCGCATTACGTTTGGTGGACC-BHQ1-3′.

For the in vivo mRNA stability assay, healthy BALB/c mice received a single intramuscular (i.m.) injection of the SV-mRNA vaccine. Serum was collected at 6, 24, and 72 h post-injection. Injection-site muscle, draining lymph nodes, liver, spleen were harvested, snap-frozen, and subjected to total RNA extraction as above. RT-qPCR was performed using primers and a probe targeting the codon-optimized antigen sequence of the SV mRNA:

- forward primer, 5′-CTGGAACCGGACCAGAATTAG-3′;
- reverse primer, 5′-GGACTCACACCATAACACTTGA-3′;
- probe, 5′-FAM-TCCGTGCTCTACAACTTTGCTCCA-BHQ1-3′.

### In vitro transfection and Western blot analysis

To verify the in vitro expression of the antigen, HEK 293 T cells were transfected with the formulated SV mRNA using the Lipofectamine™ MessengerMAX™ Reagent (Thermo Fisher Scientific, LMRNA003). At 24 h post-transfection, cells were harvested and lysed in RIPA buffer. The cell lysates were separated via SDS-PAGE and subsequently transferred onto polyvinylidene fluoride (PVDF) membranes. Following blocking, the membranes were probed overnight at 4 ℃ with a primary antibody targeting the SARS-CoV-2 Spike RBD (GeneTex, GTX635692) at a 1:1,000 dilution. Afterwards, the membranes were incubated with an HRP-conjugated secondary antibody (Affinity Biosciences, S0001), and the specific protein bands were visualized using an enhanced chemiluminescence (ECL) detection system.

### Live SARS-CoV-2 virus neutralization assay

Serum samples were heat-inactivated at 56 °C for 30 min. Following an initial 1:16 dilution, sera were serially diluted threefold in 96-well plates (50 μL/well). Authentic SARS-CoV-2 variants (GD108, BA.1, BA.5, XBB.1.5, EG.5.1, JN.1, and XDV) were diluted to 2000 TCID_50_/mL. An equal volume (50 μL) of the diluted virus (yielding 100 TCID_50_ per well) was added to the serially diluted sera. The virus-serum mixtures were gently mixed and incubated at 37 °C with 5% CO_2_ for 1 h. Vero cells in the logarithmic growth phase were detached, counted, and adjusted to 2 × 10^5^ cells/mL. Subsequently, 100 μL of the cell suspension was added to each well containing the serum-virus mixtures and incubated at 37 °C with 5% CO_2_. Cytopathic effects (CPE) were monitored daily, and final outcomes were determined at 7 days post-infection. The neutralizing antibody titer was expressed as the reciprocal of the highest serum dilution that protected 50% of the cells from CPE.

#### Pseudovirus neutralization assay

Sera were heat-inactivated (56 °C, 30 min) and serially diluted threefold from 1:20 in 96-well plates. Pseudoviruses bearing spike proteins of SARS-CoV-2 variants KP.3 (GM-0220PV169), NB.1.8.1 (GM-0220PV203-96 T), and BA.3.2 (GM-0220PV204-96 T) (Genomeditech, Shanghai, China) were diluted to 1 × 10^5^ TCID_50_/mL in complete medium. Pseudovirus (50 μL) was mixed with diluted sera and incubated (37 °C, 1 h). Virus control (VC; virus + medium, no serum) and cell control (CC; medium only) were included. hACE2-293 T cells were added (50 μL/well, 4 × 10⁶ cells/mL) and plates incubated at 37 °C for 48 h. Medium was removed and cells were lysed (20 μL/well lysis buffer, 15 min, shaking). Luciferase substrate (100 μL/well; Luciferase Assay Kit, LF009, GeneCopoeia) was added, and luminescence was measured with a FlexStation 3 Multi-Mode Microplate Reader (Molecular Devices). NT_50_ was defined as the reciprocal serum dilution yielding 50% RLU reduction relative to VC. Inhibition rate was calculated as:

$$1-\frac{RL{U}_{sample}-RL{U}_{CC}}{RL{U}_{VC}-RL{U}_{CC}}\times 100\%$$

* RLU_sample_: Sample well; RLU_CC_: Cell control; RLU_VC_: Virus control.

#### Detection of RBD-specific binding antibodies by ELISA

96-well microtiter plates were coated overnight at 4 °C with recombinant RBD proteins of SARS-CoV-2 variants BA.2 (Sino Biological, Beijing, China), KP.3 (Sino Biological, 40,592-V08H157), or JN.1 (AntibodySystem, EVV00342) at 1 μg/mL in 50 mM sodium carbonate buffer (pH 9.6) (100 μL/well). Plates were washed three times with PBST (PBS containing 0.05% Tween-20) and blocked with 2% BSA in PBS (37 °C, 1 h). Serially diluted sera were added and incubated (37 °C, 1 h), followed by HRP-conjugated goat anti-mouse IgG (Invitrogen, A-10668; 1:30,000 in 1% BSA-PBST; 37 °C, 1 h) after washing. TMB substrate (50 μL/well; Thermo Fisher Scientific, ZI4228631) was added and incubated for 15 min in the dark. The reaction was stopped with 50 μL stop solution (Solarbio, C1058), and OD was measured at 450 nm (reference: 630 nm) on a FlexStation 3 reader (Molecular Devices). The endpoint titer was defined as the reciprocal of the highest dilution with OD_450–630_ > 0.1 and ≥ 2.1-fold above the mean OD of blank (serum-free) controls.

#### Immunofluorescence assay for viral clearance and vaccine biodistribution

Lung tissues were collected at 5 days post-infection, fixed in 4% paraformaldehyde (PFA) at 4 °C for 72 h, and embedded in paraffin. Tissue Sects. (5 μm) were deparaffinized, rehydrated, and subjected to heat-induced antigen retrieval for 15–20 min. Sections were permeabilized with 0.5% Triton X-100, blocked with 5% normal goat serum for 1 h at room temperature, and incubated overnight at 4 °C with a primary antibody against SARS-CoV-2 nucleocapsid (N) protein (40,588-RC02, Sino Biological, Beijing, China). Sections were then incubated with Alexa Fluor 488-conjugated secondary antibody (ab150077, Abcam) for 1 h at room temperature in the dark, and nuclei were counterstained with DAPI. Fluorescence images were acquired with a digital slide scanner (Pannoramic MIDI II, 3DHISTECH) and analyzed using CaseViewer and Saiviewer software.

For vaccine biodistribution, healthy female BALB/c mice were immunized intramuscularly (i.m.). At 6, 24, and 72 h post-immunization, injection-site muscle, draining lymph nodes, liver, spleen, and lungs were harvested. Tissue fixation, embedding, and sectioning were performed as above. Sections were blocked and incubated overnight at 4 °C with an anti-SARS-CoV-2 Spike/RBD primary antibody (GenTex, GTX635792), followed by fluorophore-conjugated secondary antibody (Abcam, ab150077). Nuclei were counterstained with DAPI. Image acquisition and fluorescence quantification were performed as above.

#### Enzyme-linked immunospot (ELISpot) assay

To quantify the frequencies of antigen-specific cytokine-secreting T cells, the secretion of IFN-γ, IL-2, and IL-4 was evaluated using ELISpot assays (Mabtech). Briefly, spleens were harvested from euthanized mice, and single-cell suspensions were prepared by mechanical dissociation through a cell strainer. Splenocytes were subsequently isolated using a mouse spleen lymphocyte isolation kit (Solarbio, P8860). The isolated splenocytes were seeded at a density of 3 × 10^5^ cells/well into ELISpot plates pre-coated with capture antibodies against murine IFN-γ, IL-2, or IL-4 (Mabtech; catalog nos. 3321−4APT-10, 3441-4APW-10, and 3311-4APW-2, respectively). Cells were then stimulated with 2 μg/mL of recombinant SARS-CoV-2 RBD proteins derived from variants JN.1 (AntibodySystem Laboratories SAS, France, EVV00342), KP.3 (Sino Biological, Beijing, China, 40,592-V08H157), or XDV (AntibodySystem Laboratories SAS, EVV00345). Unstimulated wells served as negative controls, whereas wells stimulated with phytohemagglutinin (PHA) were used as positive controls. Following a 32 h incubation in a humidified incubator at 37 °C with 5% CO_2_, the plates were developed according to the manufacturer’s instructions. The resulting spot-forming units (SFU) were enumerated using an automated ELISpot reader (Mabtech IRIS™ 2, Mabtech).

#### TCR and BCR repertoire sequencing

TCR and BCR repertoire sequencing was performed by Wuhan Kangce Biotechnology. Mice were immunized with the SV vaccine, the BSCOV06 vaccine, or PBS (control) following the same immunization schedule. On day 14 after the second immunization, whole blood was collected and total RNA was extracted. Libraries were constructed using the KC-Digital™ Stranded TCR-seq Library Prep Kit for Illumina (DT0811-02, SeqHealth) and the KC-Digital™ Stranded BCR-seq Library Prep Kit for Illumina (DT0811-02, SeqHealth). Briefly, RNA was reverse-transcribed into single-stranded cDNA, which was then labeled with unique identifiers (UIDs) followed by PCR amplification of target regions and library construction. For data analysis, raw data were subjected to quality control to obtain clean data. Reads were error-corrected and deduplicated based on UIDs, then aligned to V, D, and J gene segments. Alignment results were used for downstream in-depth analysis. Immune repertoire sequencing data are available in the SRA database (BioProject: PRJNA1501630).

#### Flow cytometry analysis of splenic GC B cells and memory T cells

Splenocytes were isolated from BALB/c mice on day 14 after the second immunization using a lymphocyte isolation kit. Following Fc receptor blocking, cells were washed twice with PBS. Two panels were prepared: fully stained samples, single-stain controls, and unstained controls for each panel. For GC B cell detection, the following antibodies were used: Fixable Viability Stain 780 (BD, 565,388); Ms CD45R/B220 FITC RA3-6B2 (BD, 553,087); Ms CD95 PE-Cy7 Jo2 (BD, 557,653); Ms T/B-Cell Antigen PE GL7 (BD, 561,530). GC B cells were defined as *Live⁺ B220⁺ CD95⁺ GL7⁺*. For Tem/Tcm detection, the following antibodies were used: Fixable Viability Stain 520 (BD, 564,407); APC-Cy7 Rat Anti-Mouse CD3 (BD, 560,590); APC Rat Anti-Mouse CD4 (BD, 553,051); BV421 Rat Anti-Mouse CD8a (BD, 570,255); BV786 Rat Anti-Mouse CD62L (BD, 560,516); PE Rat Anti-Mouse CD44 (BD, 553,134). Cells were defined as follows: CD4⁺ Tem: *Live⁺ CD3⁺ CD4⁺ CD44⁺ CD62L⁻*; CD8⁺ Tem: *Live⁺ CD3⁺ CD8⁺ CD44⁺ CD62L⁻*; CD4⁺ Tcm: *Live⁺ CD3⁺ CD4⁺ CD44⁺ CD62L⁺*; CD8⁺ Tcm: *Live⁺ CD3⁺ CD8⁺ CD44⁺ CD62L⁺.* Sample acquisition was performed on a CytoFLEX flow cytometer (Beckman Coulter), and data were analyzed with the corresponding software.

#### Histopathology and pathological scoring

Lung tissues were fixed in 4% paraformaldehyde (PFA) at 4 °C for 72 h and embedded in paraffin. Sections (5 μm) were stained with hematoxylin and eosin (H&E) and digitized using a digital slide scanner (Pannoramic MIDI II, 3DHISTECH). Semi-quantitative histological scoring was performed with CaseViewer software by a pathologist blinded to experimental groups. Lung injury was scored across seven criteria: inflammatory cell infiltration, pulmonary hemorrhage, vascular thrombosis, bronchial obstruction, proteinaceous exudate, alveolar wall thickening, and lung consolidation. Each criterion was graded on a scale of 0 to 5. The cumulative score was categorized as mild (1–4 points), moderate (5–8 points), or severe (≥ 9 points); a score of 0 indicated no significant pathological damage.

#### Statistical analysis

All data are presented as mean ± standard deviation (SD). Statistical analyses were performed using GraphPad Prism 10.4.1 software. Comparisons between multiple groups were conducted using one-way analysis of variance (ANOVA) followed by Dunnett’s test for multiple comparisons. A value of *P* < 0.05 was considered statistically significant. Significance levels are denoted as follows: *P < 0.05; **P < 0.01; ***P < 0.001; ****P < 0.0001; and ns indicates no significant difference.

## Supplementary Information

## Data Availability

All the data from the corresponding authors are available upon reasonable request.
