# Supplementary material for: A dimeric mRNA vaccine based on key-mutant and KP.3 RBDs induces broad-spectrum immunity and protects mice against JN.1 and XDV challenges
Source: Mol Biomed. 2026 Sep 3;7:158. doi: 10.1186/s43556-026-00560-y (PMC13538340; doi:10.1186/s43556-026-00560-y)
Supplement: Supplementary file 1 [file 43556_2026_560_MOESM1_ESM.docx]

**A dimeric mRNA vaccine based on key-mutant and KP.3 RBDs induces broad-spectrum immunity and protects mice against JN.1 and XDV challenges**

Jiali Xu^1,3†^, Rui Peng^1†^, Longhai Yuan^1†^, Huan Zhang^2†^, Cong Tang^1^, Yanan Zhou^1^, Hao Yang^1^, Yun Yang^1^, Qing Huang^1^, Junbin Wang^1^, Hongyu Chen^1^, Wenqi Quan^1^, Baisheng Li^2*^, Qiangming Sun^1,4,5,6*,^ Shuaiyao Lu^1,3,4,5,6*^

1. Institute of Medical Biology, Chinese Academy of Medical Sciences & Peking Union Medical College, Kunming, China

2. Guangdong Provincial Center for Disease Control and Prevention, Guangzhou, China

3. Yunnan Key Laboratory of Cross-Border Infectious Disease Control and Prevention and Novel Drug Development, Yunnan University, Kunming, China

4. State Key Laboratory of Respiratory Health and Multimorbidity, Beijing, China;

5. Key Laboratory of Pathogen Infection Prevention and Control (Peking Union Medical College), Ministry of Education, Beijing, China;

6. Yunnan Provincial Key Laboratory of Vector-borne Diseases Control and Research, Kunming, China

†These authors contributed equally.

* The corresponding author:

Shuaiyao Lu, [lushuaiyao-km@163.com](mailto:lushuaiyao-km@163.com)

Qiangming Sun, [qsun@imbcams.com.cn](mailto:qsun@imbcams.com.cn)

Baisheng Li, [libsn@126.com](mailto:libsn@126.com)

**Fig.S1 Predicted immunogenic epitopes of the SV antigen.**

**Fig.S2 Agarose gel electrophoresis analysis of RNA stability and integrity across different organ tissues.**

a.Agarose gel electrophoresis of RT-PCR products of antigen RNA from lymph node, muscle, liver, and spleen at 6 h post-immunization.

b.Agarose gel electrophoresis of RT-PCR products of antigen RNA from lymph node, muscle, liver, and spleen at 24 h post-immunization.

c.Agarose gel electrophoresis of RT-PCR products of antigen RNA from lymph node, muscle, liver, and spleen at 72 h post-immunization.

**Fig.S3 Neutralization GMT fold change at 14 days after booster immunization in BALB/c and K18-hACE2 mice (relative to GD108)**

a:Neutralization fold change at 14 days after booster immunization in BALB/c mice (relative to GD108)

b:Neutralization fold change at 14 days after booster immunization in K18-hACE2 mice (relative to GD108)

**Fig.S4 Humoral immune responses and broad neutralization activity in mice after prime and at long-term post-boost immunization.**

a. Longitudinal endpoint titres of BA.2-, JN.1-, and KP.3-specific IgG in mouse sera at days 14 and 21 post-prime immunization.

b. NT50 values against a panel of SARS-CoV-2 variants (GD108, BA.1, BA.5, XBB.1.5, EG.5.1, JN.1, and XDV) in mouse sera at days 14 and 21 post-prime immunization.

c. Endpoint titres of BA.2-, JN.1-, and KP.3-specific IgG in mouse sera at day 240 post-boost immunization.

d. NT50 values against a panel of SARS-CoV-2 variants (GD108, BA.1, BA.5, XBB.1.5, EG.5.1, JN.1, and XDV) in mouse sera at day 240 post-boost immunization.

Data are presented as mean ± SEM. Statistical analysis was performed using one-way ANOVA with Dunnett's test. Significance levels: *P < 0.05; **P < 0.01; ***P < 0.001; ****P < 0.0001; ns, not significant.

**Fig.S5 Flow cytometric analysis of splenic germinal center B cells, effector memory T cells (Tem), and central memory T cells (Tcm) in BALB/c mice at 14 days post-boost immunization.**

a.Quantification and gating strategy for CD4⁺ and CD8⁺ central memory T cells (Tcm) and effector memory T cells (Tem) across groups.

b.Quantification and gating strategy for splenic germinal center B cells across groups.

**Fig.S6 Representative IFN-γ、IL-2 and IL-4 ELISpot images from the cellular immunogenicity assessment in Balb/c mice.**

**Fig.S7 Immunogenicity evaluation of SV in K18-hACE2 transgenic mice**

a.RBD-specific binding antibody titers against BA.2, JN.1 and KP.3 in K18-hACE2 mouse sera (n=10).

b.Neutralizing antibody titers against diverse SARS-CoV-2 variants in the sera of immunized BALB/c mice (n=10).

c.Representative IFN-γ、IL-2 and IL-4 ELISpot images from the cellular immunogenicity assessment in K18-hACE2 mice.

d.Quantification of antigen-specific cytokine-secreting splenocytes harvested from K18-hACE2 mice (n=3).

Data are presented as mean ± SEM. Statistical analysis was performed using one-way ANOVA with Dunnett's test. Significance levels: *P < 0.05; **P < 0.01; ***P < 0.001; ****P < 0.0001; ns, not significant.

**Fig.S8 Body temperature changes, nasopharyngeal swab viral load and lung TCID_50_ in BALB/c and K18-hACE2 mice following XDV infection.**

a.Post-infection body temperature changes in K18-hACE2 and BALB/c mice

b.Viral load in nasal swabs of BALB/c and K18-hACE2 mice.

c.Viral load in throat swabs of BALB/c and K18-hACE2 mice.

d.Survival curve of BALB/c mice after XDV infection

e.Viral TCID_50_ in the lungs of BALB/c and K18-hACE2 mice on day 5 after XDV infection.

Data are presented as mean ± SEM. Statistical analysis was performed using one-way ANOVA with Dunnett's test. Significance levels: *P < 0.05; **P < 0.01; ***P < 0.001; ****P < 0.0001; ns, not significant.

**Fig.S9 SV vaccination confers protection against the JN.1 variant in both BALB/c and K18-hACE2 mice.**

a:Body temperature changes in BALB/c and K18-hACE2 mice after infection (n = 5).

b:Percent body weight change in BALB/c and K18-hACE2 mice after infection (n = 5).

c:Survival of K18-hACE2 mice monitored over 5 days post-infection (n = 5).

d:Viral RNA loads in nasal swabs from BALB/c and K18-hACE2 mice over 5 days post-infection (n = 5).

e:Viral RNA loads in throat swabs from BALB/c and K18-hACE2 mice over 5 days post-infection (n = 5).

f:Genomic RNA (gRNA) levels in the lungs, nasal turbinates, and trachea of BALB/c and K18-hACE2 mice at 5 days post-infection (n = 5).

g:Subgenomic RNA (sgRNA) levels, representing active viral replication, in the lungs, nasal turbinates, and trachea of K18-hACE2 mice at 5 days post-infection (n = 5).

h,i:Immunofluorescence (IF) staining of lung tissues after infection (h) and quantification of viral antigen-positive area (%) (i). Green fluorescence indicates JN.1 virus antigen; blue fluorescence (DAPI) indicates nuclei. Scale bars, 200 μm. (n = 5).

j,k:Representative H&E-stained lung tissue sections (j) and corresponding histopathological scores (k) for assessment of lung injury severity after infection. Scale bars, 200 μm. (n = 5).

Data are presented as mean ± SEM. Statistical analysis was performed using one-way ANOVA with Dunnett's test. Significance levels: *P < 0.05; **P < 0.01; ***P < 0.001; ****P < 0.0001; ns, not significant.

**Fig. S10 Lung histopathological analysis of K18-hACE2 mice at 14 days after booster immunization (n=10).**

Data are presented as mean ± SEM. Statistical analysis was performed using one-way ANOVA with Dunnett's test. Significance levels: *P < 0.05; **P < 0.01; ***P < 0.001; ****P < 0.0001; ns, not significant.
